# Effects of Audiovisual Memory Cues on Working Memory Recall

**DOI:** 10.3390/vision5010014

**Published:** 2021-03-19

**Authors:** Hilary C. Pearson, Jonathan M. P. Wilbiks

**Affiliations:** 1 Department of Clinical Vision Science, Dalhousie University, Halifax, NS B3H 4R2, Canada; hl917301@dal.ca; 2 Department of Psychology, University of New Brunswick, Saint John, NB E2L 4L5, Canada

**Keywords:** memory cues, multisensory integration

## Abstract

Previous studies have focused on topics such as multimodal integration and object discrimination, but there is limited research on the effect of multimodal learning in memory. Perceptual studies have shown facilitative effects of multimodal stimuli for learning; the current study aims to determine whether this effect persists with memory cues. The purpose of this study was to investigate the effect that audiovisual memory cues have on memory recall, as well as whether the use of multiple memory cues leads to higher recall. The goal was to orthogonally evaluate the effect of the number of self-generated memory cues (one or three), and the modality of the self-generated memory-cue (visual: written words, auditory: spoken words, or audiovisual). A recall task was administered where participants were presented with their self-generated memory cues and asked to determine the target word. There was a significant main effect for number of cues, but no main effect for modality. A secondary goal of this study was to determine which types of memory cues result in the highest recall. Self-reference cues resulted in the highest accuracy score. This study has applications to improving academic performance by using the most efficient learning techniques.

## 1. Introduction

Throughout the process of perception, individual senses are stimulated, and the information is integrated in the brain. The process of combining these inputs into a single percept is known as multisensory integration e.g., [1,2] These perceptual components are affected by selective interference, and multiple modalities may combine to yield a beneficial additive effect [3]. In the context of learning, Mayer [4] coined this concept as the multimedia principle; people learn better from words and pictures than words alone. This phenomenon has been demonstrated to lead to better memory recall [5,6,7]. However, there are various ways to present multimodal stimuli. Dousay [5] compared two different ways: graphics paired with spoken words versus graphics with redundant spoken and printed words. Post-learning testing showed that individuals using graphics paired with spoken words scored higher. One likely explanation for this is that using information from the same modality impairs the learner, a phenomenon called the redundancy principle [8]. Tindall-Ford, Chandler, and Sweller [9] further supported this principle in a series of memory recall tasks. They found that audiovisual presentation was superior to unimodal presentation except when there were redundant visual stimuli (picture, visual text, and auditory text). Do and Moreland [10] extended this multimodal benefit to 3D observational learning. Participants were divided into four groups to learn about the construction of a wood-frame house: audiovisual learning (immersive virtual environment with 3D animation and narration), visual (immersive virtual environment with 3D animation), auditory (narration), and no training. The audiovisual learners had the highest recall scores for definitions given during encoding. Multimodal advantage has even been demonstrated for vocabulary learning in advanced foreign language learners [11]. 

Memory tasks have been used in past research as a test of efficiency and benefits of multisensory learning. These studies focus mostly on short-term or working memory (WM), which is a temporary store of information with a limited capacity. A memory cue is a stimulus that aids in the retrieval of a memory trace from memory [12]. Mantyla [13] explored the use of memory cues in a series of memory recall experiments. Participants were presented with two sets of random words and asked to write down one related word for each in the first set and three related words for each in the second set. The author concluded that the accuracy of recall is directly related to the amount of encoding retrieval information. In a follow-up experiment, they found that self-generated cues had higher recall accuracy than assigned cues.

A discussion of memory recall is incomplete without acknowledging cognitive load theory. This theory explains that working memory has a limited capacity, and thus, instructional design can be adjusted to reduce cognitive load [14]. Consequently, there has been a surge of research manipulating instructional design to minimize cognitive load and, in turn, improve memory recall. For example, Jiang and colleagues [15] conducted a study where stimuli were presented in three different conditions: four different sets of four identical faces (four-same condition), four different faces (four-different) shown sequentially, or four different faces presented one at a time (single). In each of these conditions, four different stimuli were encoded; however, the four-same conditions yielded the best performance. This suggests that presenting more copies of the same stimulus has a positive effect on cognitive load. These behavioral results are supported by neural responses in ventral visual areas (VVA; [15]). These studies demonstrate that the manner in which stimuli are presented (i.e., modality, sequence and quantity) each affects the encoding and in turn retrieval of information. 

Another factor that has been shown to influence information retrieval is the congruency effect. Lehmann and Murray [16] demonstrated the effect of congruency using a continuous recognition task. Three types of stimuli were shown: solely visual, visual with a semantically congruent sound, and visual with a semantically incongruent sound. When participants were asked to indicate which objects they had not seen before, performance was optimized under conditions when a visual stimulus was accompanied by a congruent sound stimulus. Similar results have been demonstrated by considering congruency between pictures and written words [1], effects of auditory and visual stimuli on drawings [17], and detecting the direction of motion [18]. 

There remains a debate in the literature as to whether multimodality in learning is beneficial because of the additive effect of two modalities or if the two modalities compete with each other. Thompson and Paivio [3] investigated the effect of the level of imagery, capability to create mental representations, on recall. They found that high imagery audiovisual words have a negative effect on recall. When words were high in imagery, they were not recalled better than single-modality words. When the word exposure time was decreased, high imagery auditory and visual imagery were recalled better than words in one modality. The researchers concluded that this effect could have been a result of the words being presented visually and therefore redundantly, causing interference for each group with visual imagery [3]. This finding demonstrates the fact that the interference found was within modality rather than cross-modal interference. 

A retrieval model often associated with multimodal stimuli involves the functional independence of memory codes [3]. This refers to the fact that encoding information about the same stimulus from multiple modalities allows independent modality access to retrieval of memory trace. Some variations of this model state that a stimulus is perceived as a whole, but various components of the memory trace are accessed separately, while others assume that the components function as single encodings of the same stimulus [3]. The latter paradigm alludes to the interference effect. Input from both modalities functions as single encodings from the same stimulus to form a meaningful percept. 

The current research addresses an additional aspect of memory retention, types of cues that are effective for encoding. Rogers, Kuiper, and Kirker [19] addressed this idea by presenting participants with one of four question cues and then an adjective. Participants responded yes or no according to the question cue asked and were later asked to complete a free recall task of the adjectives used. The four types of question cues used were structural (i.e., physical characteristics, such as size and font), phonemic (i.e., rhyming), semantic (i.e., meaning), and self-reference (i.e., having to do with one’s self). They found that the self-reference cues yielded significantly higher recall. It is postulated that the strength of a memory cue is dependent on the depth of processing, elaborative processing, and previous experiences/associations involved [19]. Self-reference cues inherently have many experience/associations which would enhance their effectiveness as a memory cue. 

The current study investigated the effect that multisensory memory cues have on memory recall—specifically employing audiovisual memory cues. The purpose of the study was to examine the individual and combined effects of number and modality of memory cues on subsequent recall of words. Participants were asked to generate memory cues in different modalities and generate a different number of memory cues per word. They were then presented with the memory cues they created, in the same modality they were generated, and asked to determine what the original word associated with that cue was. Past research has shown that presenting more information in the same manner for encoding leads to better recall [15]. Based on previous knowledge about the creation and use of memory cues, we had three hypotheses, as follows:

**Hypotheses H1** **(H1).** *Our first hypothesis was that the use of three self-generated memory cues would yield superior recall to the use of one self-generated memory cue. This hypothesis was based on the importance of distinctiveness of memory cues, as previously described* [20,21].

**Hypotheses H2** **(H2).** *Our second hypothesis was that the use of multimodal memory cues would result in a higher recall, in line with the multimedia principle* [4].

**Hypotheses H3** **(H3).** *Our third hypothesis was that self-referential cues would yield the highest levels of recall due to their distinctiveness and high level of experiences/associations that can function as retrieval cues. To complete this analysis, we categorized memory cues into self-reference, phonemic, contextual semantic, and definitional semantic cues to evaluate for accuracy (see Table 1 for examples of these types of cues).*

## 2. Method

### 2.1. Participants

A priori power calculations were conducted using G*Power [22,23] to determine an appropriate sample size. We decided that a medium effect size (*d* = 0.5) would be the effect size of interest, with *a* = 0.05, and a desired power (1 − B) = 0.95, which yielded a desired minimum sample size of 28 participants. In order to allow for even counterbalancing of conditions and word lists, we decided to collect data from 36 participants for the study. Thirty-six Mount Allison University students registered in an Introductory Psychology course participated in this experiment in exchange for partial course credit. The ages of participants ranged from 17 to 24, with a mean age of 18.91 (*SD* = 1.48). Twenty-seven participants identified as female and nine identified as males; all reported normal or corrected to normal vision and hearing. The total testing time for each participant was approximately one hour. Seven additional participants participated in pilot testing for the stimulus sets, and data from one additional participant were excluded, as they were unable to complete the task in the time allotted. The research protocol for this project was approved by the Research Ethics Board of Mount Allison University on November 8, 2016 (Project Code 2016-051).

### 2.2. Materials

Randomized word lists were created using the MRC Psycholinguistic Database [24]. The words on the list were all two to three syllables of high concreteness (450–550 out of 700) and high familiarity (500–700 out of 700). The word length ranged from four to eight letters, and they were all high-frequency words (Freq KF: 100–10000). This ensured that there would not be words that participants were unfamiliar with and could therefore not make effective memory cues for (as they would not possess any semantic/episodic associations with words they had never seen). In total, 120 words were selected and randomly separated into 6 lists of 20 words (see Table 2 for full list of words).

### 2.3. Procedure

Testing took place in a quiet, isolated room, with only the participant and the researcher in the room together. Participants completed a consent form and a demographic questionnaire including questions about age, gender, and vision and hearing. A computer monitor located approximately 60 cm from the participant displayed each word sequentially, from the randomly assigned word lists, centrally on the screen in a black Arial font, size 48. SuperLab 5.0 (V. 2.02; Cedrus Corporation; San Pedro, CA, USA) controlled stimulus presentation. This was a within-subject study with every participant completing all six conditions: one visual cue, three visual cues, one auditory cue, three auditory cues, one audiovisual cue, and three audiovisual cues. Presentation condition and wordlist were counterbalanced across the participants in the study in order to ensure that there was no effect of specific words in a wordlist on performance. Each word was presented against a white background until the participant responded, or for a maximum of 20 s. Participants were told that a memory cue was an associated word that would spark their memory of the original word and that they were generating the cues to see what words would be commonly associated with the base word. They were asked to generate one or three cues for every word presented, depending on the condition, which was counterbalanced across participants. Regardless of the number of cues being generated, participants had a maximum of 20 s to generate them. The researcher was present and recorded all verbal memory cues generated by the participant manually, while written memory cues were entered into the computer program.

A non-verbal distraction task was completed after the participant had completed all six blocks. It consisted of a series of simple math questions including addition, subtraction, multiplication, and division with 56 questions. This task was used to ensure that participants were not rehearsing the to-be-remembered words. In doing this, we ensured that we were measuring which types of cues (self-referent vs. semantic definitional; multisensory vs. unisensory) aid in retrieval of the target word.

After completing the non-verbal distraction task, participants were given a recall task. This consisted of participants being presented with the cues that they had generated in the encoding condition. Each self-generated cue was presented either visually on a cue card, read aloud by the researcher, or a combination of both. The visual memory cues were displayed for approximately 20 s, and the auditory cues were verbally communicated once but could then be repeated as many times as the participant wanted within 20 s. The audiovisual cues were presented on a cue card and read aloud by the researcher. These cues were presented in the same order as they had been generated. Participants were asked to write down the original word associated with the memory cue(s) presented to them. Participants were debriefed, and the nature of the study was explained.

## 3. Results

**Figure 1:**
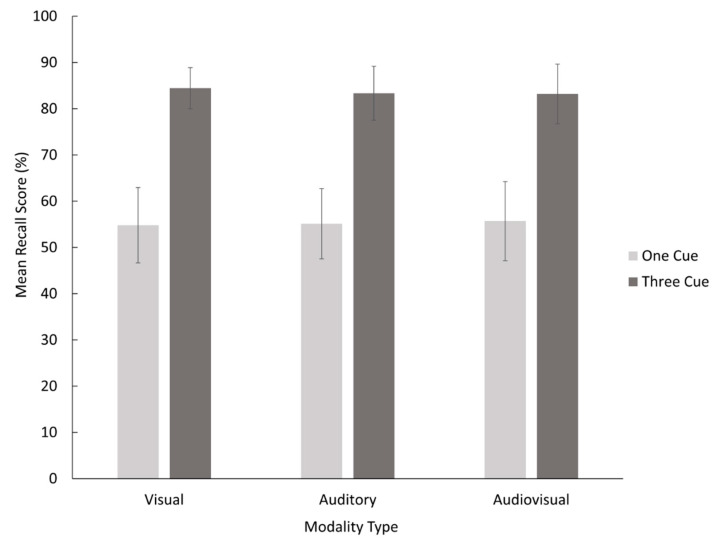
Mean recall scores by number of memory cues and modality type. Error bars represent standard errors.

Data were tabulated based on number of correct responses per participant per condition, resulting in a mean recall score as a percentage. The mean and standard errors for each condition were calculated and are presented in Figure 1. Statistical analysis was completed using SPSS (V. 25.0). A within-participants factorial ANOVA was conducted with factors of number of cues (2: one, three) × modality (3: Visual, Auditory, Audiovisual) to compare the mean recall scores. This ANOVA was used in order to analyze the effects of number of cues and modality of cues individually, as well as the interaction between those effects.

### 3.1. Number of Cues

The analysis of variance revealed a significant main effect for number of cues, *F*(1,35) = 120.57, *M_SE_* = 0.034, *p* < 0.001, $\eta_{p}^{2}$ = 0.775, which means that Hypothesis 1 that the use of three self-generated memory cues would yield a higher recall than one memory cue was supported.

### 3.2. Modality of Cues

There was no main effect for modality, *F*(2,70) = 0.04, *M_SE_* = 0.009, *p* = 0.966, $\eta_{p}^{2}$ = 0.001. Therefore, Hypothesis 2 that the use of multisensory memory cues would result in a higher recall than unisensory memory cues was not supported. The interaction between number and modality of cues was also not significant, *F*(2,70) = 0.211, *M_SE_* = 0.010, *p* = 0.811, $\eta_{p}^{2}$ = 0.014. 

### 3.3. Modes of Memory Cues

Similar to [19], the current study also aimed to determine which type of cue is most effective for recall. However, we measured how many self-generated memory cues of each type were used and their accuracy rather than providing them. The modes of memory cues we chose to analyze were based on the findings of [19]: Self-reference (i.e., autobiographical), phonemic (i.e., rhyming), and semantic. In categorizing, the cues we chose to use more specific semantic cues to avoid subjectivity: semantic definitional (i.e., antonym) and semantic contextual (i.e., fill in the blank). For examples of each type of cue, see Table 1. Rogers et al. [19] included structural question cues (the physical properties of a word—capital letter, long, etc.) in their study and found that they were not a useful cue for recall. For the current study, we had participants create memory cues by typing them, speaking them aloud, and a combination of both. Based on the fact that we cannot evaluate the structure of an auditory cue and the non-significant results of Rogers et al. [19] for structural question cues, we did not evaluate for efficiency of structural memory cues.

**Figure 2:**
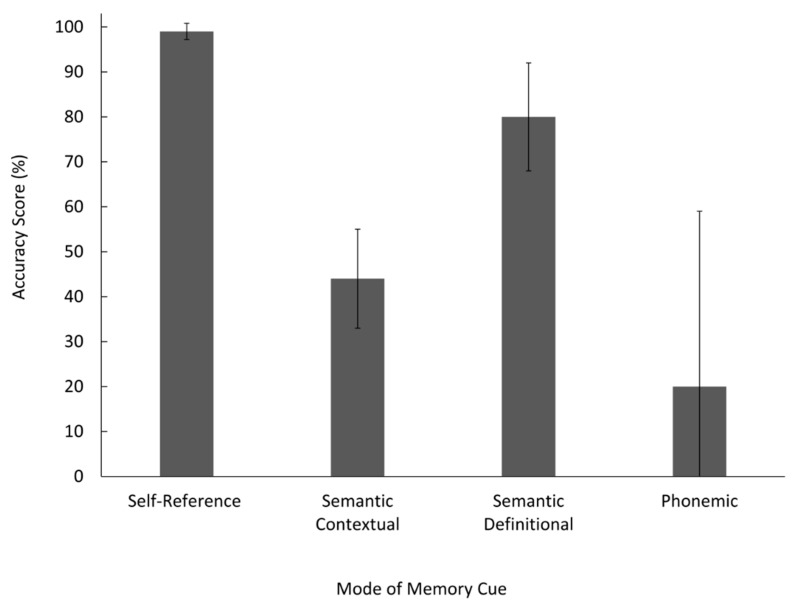
Accuracy score for each mode of memory cue condition. Error bars represent 95% confidence intervals.

Means and confidence intervals were calculated for each mode of memory cue, and effect sizes were calculated between each mode of cue. This analysis was necessary because not all participants used all types of cues, and as such, we were unable to use traditional statistical tests (e.g., one-way ANOVA). Error bars in Figure 2 display no overlap between the 95% confidence intervals between self-reference and semantic contextual, semantic contextual, and semantic definitional, or self-referent and semantic definitional. Self-reference cues were the most effective mode of memory cue and resulted in a higher accuracy score than both semantic definitional, *r*^2^ = 0.404, *M* = 0.80, *N* = 25, and semantic contextual cues, *r*^2^ = 0.772, *M* = 0.99 *N* = 33, supporting our hypothesis. Semantic definitional cues resulted in a higher accuracy score than semantic contextual, *r*^2^ = 0.502, *M* = 0.80, meaning semantic contextual cues were the least effective mode of memory cue, *M* = 0.20.

## 4. Discussion

The main purpose of this study was to investigate whether multimodal learning was more effective than single modality learning. Additionally, we were interested in whether the use of multiple self-generated memory cues was more effective than one cue, as well as which types of cues were the most effective for recall. The first hypothesis that the use of three self-generated memory cues would yield a higher recall than one memory cue was supported, replicating the findings of Mantyla [13]. This effect has been demonstrated in other studies using different instructional design. For instance, Jiang et al. [15] found that presenting four identical faces simultaneously yielded better results on a discrimination task than presenting one lone face. Multiple stimuli presented at encoding led to better performance with both memory cues (three vs. one) and photographs.

The hypothesis that the use of multiple multisensory memory cues would result in better recall than a single unisensory memory cue was not supported. Past research in the field of audiovisual integration has demonstrated that recall and discrimination is significantly improved in picture-sound conditions than single modality conditions (e.g., [3,10,25,26]). This effect, however, did not appear in this study with the use of memory cues. Perhaps, the use of an immersive narrative virtual [10] or full color photos paired with spoken words [5,6,7] functions as more powerful audiovisual encoding stimuli than simply written and spoken words. Previous studies have found that redundant visual and auditory text does not lead to superior recall than unimodal presentation [5,9]. It is possible that the pairing of written and simultaneous auditory text, although two different modalities, was redundant and therefore less effective. This integration may only occur in visual and auditory stimuli that are dissimilar (text and pictures). However, these studies attributed this finding to the redundancy of visual text and picture paired together, not the visual and auditory text perse.

One explanation for the lack of modality effect observed during the encoding phase may be that this study did not involve articulatory suppression. Articulatory suppression involves asking a person to articulate irrelevant information while simultaneously completing a verbal task, thus preventing subvocal rehearsal [27]. It is possible that for the visual blocks, participants could have articulated the word in their head while taking in the visual stimuli. This “inner speech” would make a visual block effectively function as an audiovisual block as they are processing both visual and auditory, although internal, stimuli. The results from the factorial ANOVA indicated no significant difference between the visual blocks and the audiovisual blocks, which suggests that participants may have been subjected to both audio and visual stimuli in both blocks. Similarly, with the auditory blocks, participants could have visualized the word in their minds. This limitation could be rectified by asking participants to speak aloud non-words while thinking of their cues for visual blocks and scribbling on a piece of paper for auditory blocks. These two adjustments would keep the visual and auditory blocks under strictly unisensory conditions. Finally, our third hypothesis that self-reference cues would yield the highest recall was supported, parallel to the findings of Rogers et al. [19]. When the means, effect sizes, and confidence intervals were calculated, self-reference cues were found to be substantially more effective than semantic definitional and semantic contextual cues. There was an extremely low error rate for this type of memory cue, indicating that they almost always lead to accurate recall, supporting the results of Rogers et al. [19]. A plausible explanation for this is that self-reference cues are highly distinctive [13]. By distinctive cues, we refer to the fact that they represent unique information features that differentiate the cue from previous experimental encodings. The rationale behind this phenomenon was that participants encode the to-be-remembered word in terms of the cue, associating it more strongly. Since acoustic cues do not use elaborative rehearsal, they do not form as strong of an association. This finding is further supported by a concept Mayor [4] called the concretizing principle. It states that people learn better when unfamiliar material is presented in a way that relates it to the learner’s familiar knowledge. Overall, conceptual cues had a more distinct semantic tie in participants’ memory and were therefore better recalled.

Hunt and Worthen [28] suggest that distinctiveness has such a strong effect on memory because it requires additional processes. These supplementary processes are employed because they attract more attention due to their uniqueness, bizarreness, or salience in memory. A study conducted by Hunt and Smith [29] had participants generate cues that were similar or different from the previous blocks. The results indicated that the difference cues, which were considered distinctive processing, led to higher recall than similarity cues for both self-generated and given cues. The self-generated distinctive cues, however, in comparison to given distinctive cues were more effective. This offers a clear explanation as to why self-reference cues function so effectively as memory cues. Self-reference cues are directly related to the memories and experiences of the participant. Every individual has their own unique experiences, and therefore, these self-reference cues are highly distinct in memory.

Although there was no main effect of modality on memory recall, there are several applications to studies in this field. Academic performance heavily relies on the manner in which material is presented to students, and how students encode information themselves. This study sheds light on whether presenting information in one modality or in multiple modalities leads to the highest retention of material. A study done by Lalwani, Lwin and Ling [30] found that the presentation of advertisements in audiovisual form leads to more favorable outcomes. When they presented an advertisement for an item in both modalities, consumers’ attention increased. This effect was only present when the music/speaking was congruent with the visual stimuli presented. This finding not only supports the idea of multimodal advantage as Lehmann and Murray [16] found, but it also supports the research of Chen and Spence [17] demonstrating that congruency in both modalities can enhance performance.

The current study did not find an advantage for multisensory memory cues over unisensory memory cues. This suggests that not all the effects associated with multisensory object discrimination function the same with multisensory memory cues. Perhaps the beneficial additive effects of redundancy observed in studies such as Jiang et al. [15] are only applicable to object discrimination and not word lists. Perhaps the multimodal advantage that Seitz, Kim, and Shams [26] demonstrated in their perceptual discrimination task does not translate to audiovisual memory cues. However, a limitation of this study was that articulatory suppression was not used. A follow-up study incorporating the manipulation of articulatory suppression may manifest an effect for an advantage of multisensory memory cues. Additionally, it would be important for future research to disentangle effects occurring at different stages of the memory process. That is to say, when we analyze effects of memory cues, we need to identify whether we are testing the effects of varying conditions at encoding (e.g., [15]) or during retrieval (e.g., [3]). One way of doing this would be to run an experiment similar to the one reported here, but always present a single cue during the retrieval phase, regardless of condition (we thank an anonymous reviewer for this suggestion). In that case, for the three-cues condition, only one of those cues (chosen randomly) would be presented as a retrieval cue. If a significant difference were observed between one-cue and three-cue conditions in that experiment, this could be incontrovertible evidence that the effect of cue number was occurring at encoding (while cues were being created) and not being affected by the number of cues at retrieval.

Research on this topic merits further investigation. Potential future studies could include but not be limited to a replication of this study but with imaginative cues, or a more delayed recall task (a couple of days) testing more long-term memory. Future research should also include a manipulation of number of cues specifically at encoding and/or retrieval phases in order to disambiguate the stage at which the observed effect is occurring. Finally, it is important to note that participants in this study were recruited from undergraduate psychology classes at a university in Canada, which limits the generalizability of the results. While we do not expect there to be cultural or other demographic differences on a phenomenon such as memory, it is still important to note these concerns with regard to diversity, and to seek more widely recruited participants in future. We did, however, find a very strong effect of number of cues, indicating that the use of three memory cues rather than one is much more effective for memory recall. What is unclear at present is whether this effect occurs due to the creation of multiple cues at encoding or the presentation of multiple cues at retrieval. We also demonstrated the usefulness of self-reference memory, as it led to almost perfect accuracy scores.

## Figures and Tables

**Table 1:** Examples of possible memory cues based on presented word.

	Self-Reference	Phonemic	Semantic Contextual	Semantic Definitional
Cue	Friend	Walk	Back	Cold
Example Response	Sarah	Talk	Space	Hot

**Table 2:** List of words used per block.

List 1	List 2	List 3	List 4	List 5	List 6
INDUSTRY	CORNER	SQUARE	WELL	PRIZE	SHAPE
COUNTRY	DATE	MORNING	CLUB	DRINK	DISEASE
AUDIENCE	SHOT	GROUP	FIGURE	STORM	FLASH
STUDENT	VOICE	WALK	PARTY	NOVEL	SHOP
SOUND	COURT	NIGHT	NOTE	SALARY	BREATH
TEST	FAMILY	PEOPLE	ESSAY	JUDGE	INCENSE
MUSIC	ARMY	COLD	BEVERAGE	ADULT	CHART
POINT	SPRING	WHITE	GRAVE	SUIT	GANG
TOOL	LIGHT	RACE	CRUMB	HOLE	SHOUT
HUSBAND	PLANE	STAFF	CROSS	CAPITOL	CITIZEN
MEMBER	MATERIAL	FRIEND	HOCKEY	PARTNER	GERM
STAND	HOME	BLUE	FOOTSTEP	SHIVER	BIRTH
BILL	PATTERN	WORLD	MESSAGE	SCREAM	GUEST
STEP	PLAY	BACK	MOLD	MAGNET	MAIL
WRITING	CASE	LINE	CASH	COUSIN	SHADOW
OFFICER	LEAD	PROPERTY	PILE	GREEN	AISLE

## Data Availability

Data available upon request from the corresponding author.
